# Panobinostat Induced Spatial In Situ Biomarkers Predictive of Anti-PD-1 Efficacy in Mouse Mammary Carcinoma

**DOI:** 10.3390/cells12020308

**Published:** 2023-01-13

**Authors:** Zuzana Tatarova, Dylan C. Blumberg, AeSoon Bensen, Gordon B. Mills, Oliver Jonas

**Affiliations:** 1 Department of Biomedical Engineering, OHSU Center for Spatial Systems Biomedicine, Oregon Health & Science University, Portland, OR 97239, USA; 2 Knight Cancer Institute, Oregon Health & Science University, Portland, OR 97239, USA; 3 Department of Radiology, Brigham and Women’s Hospital, Harvard Medical School, Boston, MA 02115, USA; 4 Department of Cell, Developmental & Cancer Biology, Oregon Health & Science University, Portland, OR 97239, USA; 5 Division of Oncologic Sciences, Oregon Health & Science University, Portland, OR 97239, USA

**Keywords:** anti-PD-1, breast cancer, immunogenic cell death, predictive biomarkers, panobinostat, spatial analysis, cancer stem cells, MIMA, immunotherapy

## Abstract

Immunotherapies, including anti-PD-1 immune checkpoint blocking (ICB) antibodies, have revolutionized the treatment of many solid malignancies. However, their efficacy in breast cancer has been limited to a subset of patients with triple-negative breast cancer, where ICBs are routinely combined with a range of cytotoxic and targeted agents. Reliable biomarkers predictive of the therapeutic response to ICB in breast cancer are critically missing, though a combination response has been associated with immunogenic cell death (ICD). Here, we utilized a recently developed integrated analytical platform, the multiplex implantable microdevice assay (MIMA), to evaluate the presence and spatial cell relations of literature-based candidate markers predictive of ICB efficacy in luminal mouse mammary carcinoma. MIMA integrates (i) an implantable microdevice for the localized delivery of small amounts of drugs inside the tumor bed with (ii) sequential multiplex immunohistochemistry (mIHC) and spatial cell analysis pipelines to rapidly (within days) describe drug mechanisms of action and find predictive biomarkers in complex tumor tissue. We show that the expression of cleaved caspase-3, ICAM-1, neuropilin-1, myeloperoxidase, calreticulin, galectin-3, and PD-L1 were spatially associated with the efficacy of panobinostat, a pan-HDAC inhibitor that was previously shown to induce immunogenic cell death and synergize with anti-PD-1 in breast cancer. PD-L1 by itself, however, was not a reliable predictor. Instead, ICB efficacy was robustly identified through the in situ hotspot detection of galectin-3-positive non-proliferating tumor zones enriched in cell death and infiltrated by anti-tumor cytotoxic neutrophils positive for ICAM-1 and neuropilin-1. Such hotspots can be specifically detected using distance-based cluster analyses. Single-cell measurements of the functional states in the tumor microenvironment suggest that both qualitative and quantitative effects might drive effective therapy responses. Overall, the presented study provides (i) complementary biological knowledge about the earliest cell events of induced anti-tumor immunity in breast cancer, including the emergence of resistant cancer stem cells, and (ii) newly identified biomarkers in form of specific spatial cell associations. The approach used standard cell-type-, IHC-, and FFPE-based techniques, and therefore the identified spatial clustering of in situ biomarkers can be readily integrated into existing clinical or research workflows, including in luminal breast cancer. Since early drug responses were detected, the biomarkers could be especially applicable to window-of-opportunity clinical trials to rapidly discriminate between responding and resistant patients, thus limiting unnecessary treatment-associated toxicities.

## 1. Introduction

The immune system has been shown to be actively involved in suppressing the initiation and progression of cancer, and thus therapeutic modifications of immune-related events are increasingly utilized as the fourth pillar in cancer treatment, along with surgery, chemotherapy, and radiotherapy [1,2,3]. Specifically, the regulation of the immune system through immune checkpoint blockade (ICB), most notably via cytotoxic T-lymphocyte-associated protein 4 (CTLA-4) and programmed death-1 (PD-1), is the most clinically advanced type of immunotherapy and is approved for multiple cancers. Both the CTLA-4 and PD-1 pathways act to prevent the hyperactivation of the immune system, and the inhibition of these inhibitory molecules has been shown to be capable of mediating robust immune activation against cancer. As of 2018, when Drs. James Allison and Tasaku Honjo received a Nobel prize for their seminal work on ICB, 20–40% of cancer patients experienced tumor regression with PD-1 pathway blockade [4]. In many indications, however, ICBs are mostly ineffective as a monotherapy and must be combined with cytotoxic or targeted agents to achieve a significant clinical benefit. A fraction of these patients remain unresponsive, and thus ICB-associated mechanisms continue to be included in extensive investigations in current cancer research.

### 1.1. Immune Checkpoint Blockade and Its Clinical Biomarkers in Breast Cancer

While ICB is a feasible option for patients with melanoma and lung cancer, patients with so-called immunologically cold tumors (including breast cancer (BC)) benefit less [5]. Reduced sensitivity to ICB has been associated with low tumor-infiltrating lymphocyte (TIL) counts [6]. However, TIL counts have been shown to have inconsistent prognostic and predictive value in BC. For instance, in adjuvant and neoadjuvant chemotherapy responses, they correlate with survival benefit in human epidermal growth factor receptor 2 (HER2)-positive breast cancer and triple-negative breast cancer (TNBC) [7,8], whereas in luminal HER2-negative BC increased TILs are an adverse prognostic factor for survival [8]. Among other biomarkers, the expression of the immune evasion molecule programmed death-ligand 1 (PD-L1) and tumor mutational burden have been associated with favorable efficacy of ICB in a small subset of TNBC patients [9]. The IMpassion130 clinical trial was one of the first studies to suggest this, showing that the combination of atezolizumab (anti-PD-1) and nab-paclitaxel was beneficial in patients stratified according to PD-L1 expression [10]. However, this finding did not hold up in subsequent studies [11]. Thus, multiple open questions arose defining the use of ICB in BC, including appropriate TIL and PD-L1 expression assessment, the selection of the ideal chemotherapy partner to maximize therapeutic efficacy, other biomarker discovery for patient stratification, and their predictive value in luminal tumors, where late recurrences are common. Adding the possible risk of immune-related adverse events and the high costs of ICB, the discovery and implementation of novel biomarkers to guide patient benefits are critically required.

### 1.2. Immunogenic Cell Death Sensitizes Tumors to ICB Efficacy

Immunogenic cell death (ICD) is a form of cellular death that can trigger a long-term protective anti-tumor immune response in an immunocompetent host. ICD can be induced by chemotherapeutics [12,13], targeted anti-cancer agents [1,14], physical modalities such as radiotherapy [15], oncolytic viruses [16], and gaseous molecules [17]. One hallmark of ICD is a spatiotemporal release of danger-associated molecular patterns (DAMPs) from dying cells that recruit antigen-presenting cells that ultimately prime a T-cell-mediated immune response [18]. Interventions inducing ICD can induce T-cell infiltration [1,17]. Given that tumors need to be pre-infiltrated by T cells for ICB efficacy, interventions inducing ICD could synergize with the PD-1 blockade pathway. While it is not the only mechanism essential for effective T-cell-mediated responses—antigenicity [1] and permissive TME have equally important complementary roles [19,20]—ICD is thought to be a key determinant of immune surveillance [20]. Several molecular events are associated with ICD in vitro [14,18], including the translocation of calreticulin molecules to the cell surface [21], adenosine triphosphate (ATP) secretion [12], type I interferon (IFN) and nuclear factor kappa-light-chain-enhancer of activated B cells (NF-κB) [22] signaling, the passive and acute release of the nuclear high mobility group box 1 (HMGB1) protein [23], and the co-release of CXCL1, CCL2, and CXCL10 [24]. Bona fide ICD is defined in whole animals, typically with a prophylactic vaccination of immunocompetent mice, where dead cells provide immunological protection against a subsequent rechallenge with live cells of the same type. While the molecular determinants of ICD are currently well described; the cellular events in situ are still poorly understood [1,12], where in situ means that the measurement is taken in the same place where the phenomenon is happening without isolating it from the surrounding environment. Secondly, DAMP markers have been tested as biomarkers of ICD induction, but so far have not allowed the identification of interventions that cause immunogenic cell death in vivo and synergize with anti-PD-1 [25,26].

### 1.3. Panobinostat Was Predicted to Induce ICD and Synergize with Anti-PD-1 in Breast Cancer Using a Multiplex Implantable Microdevice Assay System

Multiplex imaging technologies on single formalin-fixed, paraffin-embedded (FFPE) tissue samples have been transformative in understanding the biology and heterogeneity of cancers by studying single cells in their native spatial contexts. Along with methods for high-dimensional data integration, their application increasingly improves the diagnosis and treatment of cancer [27,28,29,30]. We recently developed an integrated technological and computational tool, termed the multiplex implantable microdevice assay (MIMA), for the efficient assessment of the tumor microenvironmental effects of multiple drugs and drug combinations. The MIMA system deploys a multiwell intratumoral drug delivery device [31] and multiplex immunohistochemistry (mIHC) staining and imaging [1,27,28] for the evaluation of the presence of 30+ proteins within single cells in an XY-coordinate space. An integrated cell classification approach introducing “standard cell types” and the computational analysis of spatial cell associations with a local drug stimulus rapidly defines the drug mechanism of action and predicts which conventional anti-cancer agents synergize with specific immune-based therapies, including anti-CSF1R, anti-CD40, and anti-PD-1 immunotherapies [1]. In a small-scale screening using seven FDA-approved drugs in a genetically engineered mouse model of breast cancer, we previously described the earliest cellular events of induced anti-tumor immunity and found a pan-histone deacetylase (HDAC) inhibitor, panobinostat, that induces ICD, recruits CD8 T cells inside the tumor bed, and synergizes with the anti-PD-1 monoclonal antibody to decrease the tumor burden [1].

In this study, we provide a literature review of protein-level IHC-based biomarkers (rather than, e.g., soluble or in vitro biomarkers) that are directly or indirectly associated with induced anti-tumor immunity, immunogenic cell death, TIL increase, and/or PD-1 pathway blockade efficacy, independent of breast cancer. We measured whether these biomarkers are specifically enriched at the panobinostat delivery site using the MIMA system in the MMTV-PyMT (mouse mammary tumor virus–polyoma middle T antigen) model of breast cancer. Displaying the candidate-marker-positive cells in an XY-coordinate space, we show that cleaved caspase-3 (CC3), intracellular adhesion molecule 1 (ICAM-1), myeloperoxidase (MPO), galectin-3, neuropilin-1, calreticulin, and PD-L1 were specifically enriched at the local panobinostat-induced ICD site compared to control (random intratumoral) regions. We quantified these enrichments by measuring biomarker expression per cell as a function of the distance from the well, splitting the assay area into immediate, proximal, border, distal, and remote “zones” and measuring biomarker expression on standard cell types. This semi-supervised approach helped us to identify about 70 novel marker combinations that significantly and uniquely appeared with the local panobinostat stimulus. A distance-based cluster analysis showed them to co-occur, with clustering parameters mapping at least 10 cells within a maximum distance range of 30 to 75 μm. This overall approach has the potential to (i) define the cellular role of the newly studied biomarkers in the therapy response and (ii) identify hotspots in whole FFPE tumor tissues or biopsies that could be used for patient stratification in clinical practice. We summarize the approach and provide illustrative examples in the following sections.

## 2. Materials and Methods

### 2.1. Murine Models and Studies

MMTV-PyMT mice were from Dr. Lisa Coussens and were purchased from the Jackson Laboratory. All animal studies were conducted in accordance with protocols approved by the institutional animal care and use committee (IACUC) at OHSU (protocol number: IP00000956). All mice were bred and housed in specific-pathogen-free conditions under a standard 12 h light/12 h dark cycle. Virgin female mice at 12–15 weeks of age were used for all experiments.

The mice were monitored daily to determine any possible effects on the general condition of the animals using parameters established by (Morton and Griffiths, 1985). The guidelines for pain, discomfort, and distress recognition were used to evaluate weight loss, appearance, spontaneous behavior, behavior in response to manipulation, and vital signs. All implanted mice were well conditioned (BC score: 3); no signs of pain, discomfort, or distress were observed.

### 2.2. Microdevice Implantation and Sample Collection

The nanodose drug delivery devices were cylindrical microdevices 5.5 mm in length and 750 μm in diameter manufactured from medical-grade Delrin acetyl resin blocks (Mitsubishi Chemical Advanced Materials, Lenzburg, Switzerland) by micromachining (CNC Micromachining Center, Cameron Micro Drill Presses, CA, USA) with 18 reservoirs measuring 200 μm (diameter) × 250 μm (depth) on the outer surface. The reservoirs were packed with panobinostat mixed with a polyethylene glycol (PEG, MW 1450, Polysciences, Warrington, PA, USA) polymer at 20% concentrations. The microdevices were implanted for three days in MMTV-PyMT mice with late-stage spontaneously growing tumors in all experiments. The tumor size was between 1.2 and 1.5 cm in the longest dimension at the time of implant. Tumors were excised at three days after device implantation, fixed for 48 h in 10% formalin or 4% paraformaldehyde, then perfused with paraffin. Specimens were sectioned using a standard microtome, and 5 μm tissue sections were collected from each reservoir. Dry FFPE tissues were baked in a 65 °C oven for 30 min. Following deparaffinization with xylene and rehydration in serially graded alcohol to distilled water, slides were subjected to endogenous peroxidase blocking in fresh 3% H_2_O_2_ for 10 min at RT. Sections were then stained using multiplex immunohistochemistry with a mouse-specific antibody panel [1].

### 2.3. Multiplex Immunohistochemistry (mIHC)

mIHC consisted of iterative cycles of (i) staining, (ii) whole-slide scanning, and (iii) the heat and chemical stripping of antibodies and chromogen. First, the slides were subjected to staining with F4/80 and CSF1R antibodies (cycle zero, no antigen retrieval) and hematoxylin staining (S3301, Dako, Agilent, Santa Clara, CA, USA) for 1–5 min, followed by whole-slide scanning. The slides were then subjected to the first heat-mediated antigen retrieval in 1× pH 5.5–6 citrate buffer (Biogenex Laboratories, Fremont, CA, USA, HK0809K) for 90 s in a low-power microwave and 16 min in a steamer, followed by protein blocking with 10% normal goat serum (S-1000, Vector Lab, Newark, CA, USA) and 1% bovine serum albumin (BP1600-100) in 1× PBS for 30 min at RT. Slides were incubated with primary antibodies (concentrations defined in [1]) for 1 h at RT or 16–17 h at 4 °C while in a dark humid chamber. The signal was visualized with either anti-rabbit or anti-rat Histofine Simple Stain MAX PO horseradish peroxidase (HRP)-conjugated polymer (Nichirei Biosciences, Tokyo, Japan), followed by peroxidase detection with 3-amino-9-ethylcarbazole (AEC). Two or three drops of HRP polymer was used for up to nickel-sized or whole-slide tissue samples, respectively. The timing of AEC development was determined by a visual inspection of the positive control tissue [1] for each antibody. All washing steps were performed for 3 × 5 to 10 min in 1× PBS while agitating. Slides were mounted with a filtered 1× PBS with 0.075% Tween20 (BP337100) using a Signature Series Cover Glass (Thermo Scientific, Waltham, MA, USA, 12460S). Images were acquired using the Aperio ImageScope AT (Leica Biosystems, Wetzlar, Germany) at 20× magnification, after which the coverslips were gently removed in 1× PBS while agitating. Within one cycle, the removal of AEC and HRP inactivation were accomplished by incubating the slides in 0.6% fresh H_2_O_2_ in methanol for 15 min; AEC removal and the stripping of antibodies were accomplished by ethanol gradient incubation and heat-mediated antigen retrieval, as described above, between cycles. After washing and protein blocking, samples were subjected to the next round of staining. The readout antibody panel was carefully designed so that it broadly captured all major TME subtypes and states [1], which helped to explain the target protein’s function and the drug’s mechanism of action.

### 2.4. Image Processing and Feature Extraction of mIHC Images

Iteratively digitized images were co-registered using (i) Matlab (The MathWorks, Inc., Natic, MA, USA, version 2019b), utilizing the detectSURFFeatures algorithm from the Computer Vision Toolbox, and (ii) the Linear Stack Alignment with SIFT plugin (Fiji) so that cell features overlapped down to the single-pixel level. Hematoxylin-stained images were color-deconvoluted for single-cell nuclear segmentation to generate a binary mask using the watershed function and standard image processing steps (noise removal, erosion, and dilation; Fiji [32]). The AEC chromogenic signal was extracted using the NIH plugin RGB_to_CMYK to separate the AEC signal into the yellow channel for improved sensitivity of IHC evaluation [1,33,34]. Gray-scale images of all proteins and the binary mask were imported to CellProfiler (version 3.1.8, Broad Institute, Cambridge, MA, USA) [35] to quantify the mean single-cell signal intensity, as defined by the mask, which was scaled to a range of 0–1. The IdentifyPrimaryObjects module was used to identify nuclei from the mask; the MeasureObjectIntensity module measured the mean intensity of each object for each protein. The mean signal intensity per cell output was imported to FCS Express 6 and 7 Image Cytometry Software (DeNovo Software, Pasadena, CA, USA) to perform multidimensionality reduction to classify “standard cell types”. Gating strategies and hierarchical cell classification are presented in Figure 4a–c. Polygonal gates moving around a central vertex without changing the polygon shapes were used to obtain quantitatively reproducible multiplex data, batch to batch, independent of the measured condition. Positive control tissues were used to help define the single-parameter threshold for positivity using manual gating. A total of 8000–18,000 cells were analyzed for feature extraction in the assay area located above the drug release site (Figure 2b). Cell phenotypes with a >1% rate are shown, as this is a typical threshold used in clinical practice to stratify patients benefiting from PD-1/PD-L1-axis-targeting therapies [36]. The experimental condition of the assay area was compared to a random control intratumoral region located perpendicular and/or far from the drug-releasing reservoir. To obtain greater control over cofounding variables, paired-sample one-tailed *t*-tests were used to determine the enrichment of induced TME states. The rate of positivity and significance are presented in the form of a heatmap or bar graphs. The quality of the single-cell data was ensured by excluding deformed (folded), lost, or unevenly stained tissue (border effects). Single-cell data from FCS Express were extracted in a data grid to Matlab for downstream spatial systems analyses. In computed images, leukocytes are presented independent of the Epcam ± status.

### 2.5. Spatial Cell Analysis In Situ

The distance-based cluster function finds clusters in a set of spatial points expressed in XY space (adapted and modified from Yann Marcon; Matlab October 2019). The clustering is based on the Euclidean distance between the points (cells). The function does not require the number of clusters to be known beforehand. Instead, each cell clusters with the closest neighboring cell if the distance between the two cells is shorter than the defined threshold. The minimum number of cells per cluster is defined by the user. The function outputs non-clustering cells in gray, while each cluster meeting the defined parameters (the minimal number of cells within the maximum distance range) is presented in randomized colors.

Clusters within the maximum defined distance merge and share one color. Treatment-specific cluster formation with a cluster definition of a minimum of 10 cells within a 50 μm distance was generalizable to all markers and standard cell types. Random circular regions with 110–220 μm diameters were selected in the proximal, border, distal, and remote zones. The numbers of cells and replicates used are presented in Figure 4e and in the figure legends.

Two-dimensional composite images were created using Fiji [32].

### 2.6. Code and Data Availability

The image analysis pipeline using publicly available functions is detailed in the Section 2 and in the Supplementary Information. All data that support the findings of this study are available within the article and its Supplementary Information or from the corresponding authors upon reasonable request.

## 3. Results

### 3.1. Literature Review to Find Biomarkers of Ongoing/Induced Anti-Tumor Immunity

We identified candidate biomarkers of induced anti-tumor immunity in breast cancer by performing a literature review focused on protein-level probes that were previously associated with ICD induction; high TILs, including CD8 T cells; inflammatory responses; and/or the response to ICB. The association could be direct or indirect (correlation), and the review was not limited to breast cancers. Both pre-treatment (prognostic) and post-treatment (predictive) biomarkers were considered. A summary of our review is presented in Table 1. We also tested the specificity of the antibodies against the target proteins using IHC in mouse FFPE samples (Supporting Figure S1).

### 3.2. Components of the Multiplex Implantable Microdevice Assay (MIMA)

**Figure 1:**
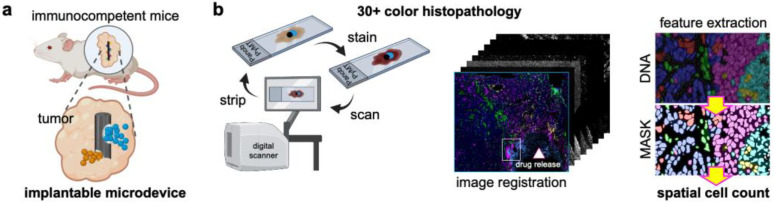
**Multiplex implantable microdevice assay (MIMA) components.** (**a**) A schematic presentation of the implantable microdevice inserted into the tumor of an immunocompetent mouse and a magnified view of two drugs released into spatially separate regions of the same tumor. (**b**) Multiplex immunohistochemistry (mIHC) on a single FFPE slide, including the drug-affected region of the tumor. mIHC uses iterative (i) staining by the deposition of chromogen, (ii) a brightfield digital scanner to image the target signal, and (iii) stripping the target signal with organic solvents (left). The images were co-registered, including the nuclear staining (DNA), which defines the mask for single-cell segmentation and the spatial cell count (right). In this way, 30+ markers could be evaluated in each individual cell.

The MIMA system integrates high-throughput and high-content components. One of them is a small nail-like implantable microdevice for the localized intratumoral delivery of 18 treatments, each at nanoliter volumes [31]. To evaluate the treatment-induced immune milieu, the microdevices were implanted into tumors of immunocompetent mice (Figure 1a). After a defined amount of time (three days in this study), the tumors were extracted from the implanted mice, formalin-fixed, and paraffin-embedded with the implanted device in place. Subsequently, the tissue was sectioned perpendicular to the device main axis, and the resulting FFPE sections were mounted on slides for further processing. The slides that include directional drug-induced changes in the tumor/tumor microenvironment were processed using the second component of MIMA, which is multiplex immunostaining followed by image processing, cell classification, and spatial single-cell analysis (Figure 1b).

The cell classification system used the single-cell expression of 30+ proteins to define the locations of specific “standard cell types” in regions near each drug delivery site. The spatial analyses of the composition and organization of these cells provided information about the delivered drug mechanisms of response. The details of the “standard cell type” classification as well as the MIMA spatial analyses are described in Tatarova et al. [1] and are partly summarized in the next sections.

### 3.3. Candidate Biomarkers Were Spatially Associated with Panobinostat Drug Release Site

**Figure 2:**
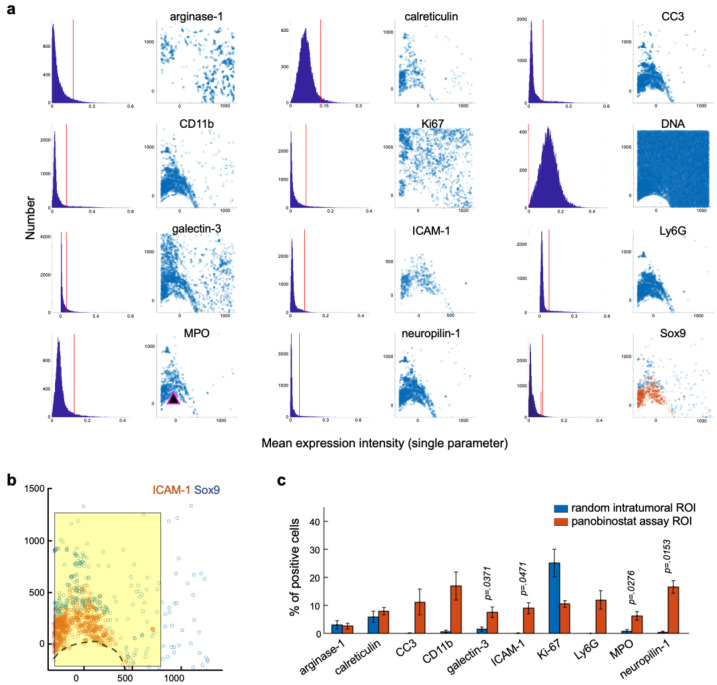
**Panobinostat induces treatment-specific infiltration of myeloid cells and enrichment of candidate ICB biomarkers.** (**a**) Marker expression in XY coordinates in the panobinostat assay area. Each dot represents a marker-positive cell, as titled above the scatter plot. The single-parameter threshold for positivity was defined by manual gating using FCS Express 6 and 7 Image Cytometry Software, visual inspection, and positive control tissue. The mean expression intensity is presented in the form of a histogram, and the threshold for positivity is depicted by a red line (left graphs). (**b**) Magnified view of ICAM-1 and Sox9 expression in XY space. Coordinate [0,0] identifies the drug source, and the direction of the drug release is upward. Graphs are shown offset to the left so that both the panobinostat assay region (yellow) and the non-affected control area are visible in the presented space. (**c**) Quantification of single-cell events inside the panobinostat assay area compared to control. Bars are means ± s.e.; *n* = 3 reservoirs from two to three MMTV-PyMT tumors. The device was implanted for three days. Significance was calculated using a paired-sample two-tailed *t*-test.

We assessed the spatial association between the candidate IHC-based biomarkers (Table 1) and induced immunogenic cell death, as revealed by the MIMA system [1]. We accomplished this by inserting microdevices loaded with panobinostat into the tumors of the MMTV-PyMT mice [55,56,57], which were reported to arise from luminal cells, cluster with the luminal B subtype using gene expression profiling, and closely mimic the heterogeneity and progression of human breast cancers [55,56,57,58]. We chose spontaneous tumors rather than tumors induced by orthotopic or subcutaneous transplantation in order to have tumor microenvironments that closely mirrored the functional role of stromal compartment evolution in de novo tumor progression in human cancers [59,60]. These MIMA studies showed that the panobinostat drug stimulus induced the recruitment of myeloid cells (CD11b), mostly neutrophils (Ly6G; Figure 2a–c) [1].

The majority of the candidate biomarkers (CC3, ICAM-1, MPO, and neuropilin-1) were spatially associated with this cellular population when presented in XY-coordinate space (Figure 2a). The presence of the “eat me” calreticulin signal extended beyond the neutrophil/death region, further confirming that panobinostat-induced cell death is immunogenic [21]. Similarly, galectin-3 appeared specifically in the panobinostat assay region (Figure 2b), but was present both within as well as beyond the cell death region. The two areas were split by the enrichment of cancer stem cells (CSCs), which, in breast cancer, we mark with the Sox9 marker [61] (Figure 2b). The quantification of the single-cell events inside the panobinostat assay area (Figure 2b) revealed that galectin-3-, ICAM-1-, MPO-, and neuropilin-1-positive cells were significantly enriched by the local panobinostat stimulus (Figure 2c).

**Figure 3:**
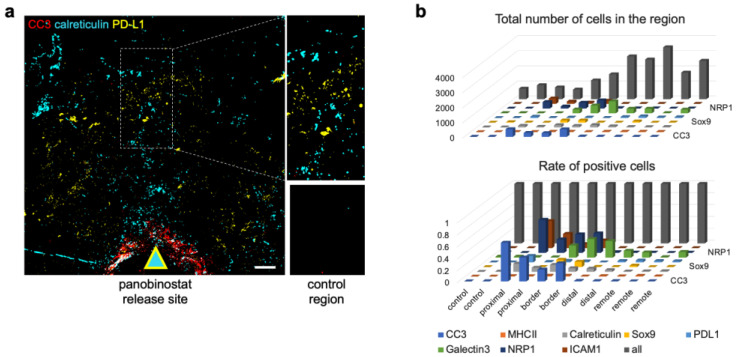
**Spatial distribution of the ICB biomarkers at local panobinostat drug-delivery site.** (**a**) A sample composite image of cell death (CC3), DAMP (calreticulin), and an established marker of ICB efficacy (PD-L1) at the panobinostat well. Control region: bottom right. Scale bar: 200 μm. The image was adapted from [1]. (**b**) Quantification of candidate biomarkers after zonal stratification, presented in the form of a 3D bar graph. Two to three replicates are presented per zone.

We were able to observe increased expression of the established ICB-response biomarker, PD-L1, in only one of three samples. This increase was distant (~1500 μm) from the well (Figure 3a). Overall, the quantification of the individual biomarkers across the space showed them to be split into “zones” with distances from the well, with (i) CC3, ICAM-1, and neuropilin-1 being located in immediate proximity to the well (*proximal* zone), (ii) CSCs being located at the outer *border* of this region, (iii) galectin-3 and calreticulin dominating the *distal* zone, and (iv) PD-L1 being located *remotely* (Figure 3b). We interpreted this to mean that the target proteins might have spatial preferences for each other and thus might be functionally associated in the panobinostat mechanism of action.

### 3.4. Cell Type Classification of the Candidate Biomarkers in Space Defines Their Roles during ICD

**Figure 4:**
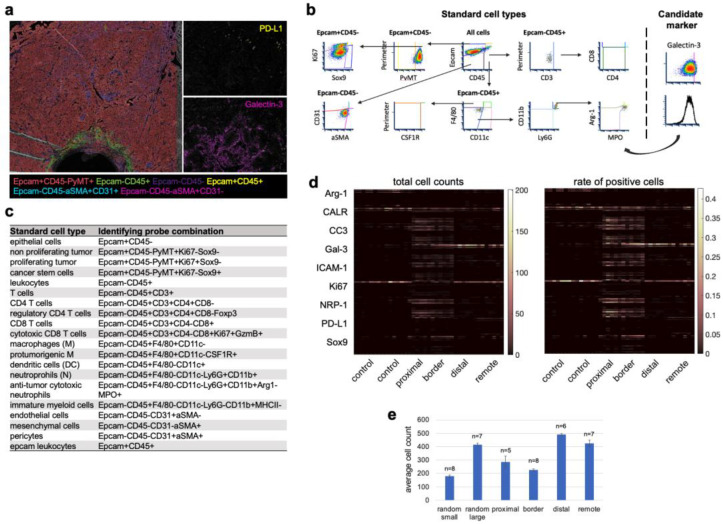
**Analytical design to quantify the cell type specificity and spatial enrichment of ICB biomarkers at the panobinostat well.** (**a**) Pseudo-colored image of a tumor sample implanted with a microdevice releasing panobinostat for three days. Marker combinations define the main tumor cell types: tumor (red), leukocytes (green), pericytes (cyan), fibroblasts (magenta). (**b**) Density plot of dimensionality reduction in hierarchical gating to define “standard cell types”. Candidate ICB biomarkers were measured within these cell subsets. The threshold for positivity was determined manually using FCS Express 6 and 7 image cytometry software and a positive control tissue. (**c**) List of probe combinations identifying standard cell types. (**d**) Heatmap of total cell counts (left) and rate of positive cells (right) at depicted panobinostat zones (x-axis). The control region was derived from the same section but in a panobinostat-non-affected area. (**e**) Bar graph showing the average cell count per analyzed zone and the number of replicates used (*n*). Bars are means ± s.e.

**Figure 5:**
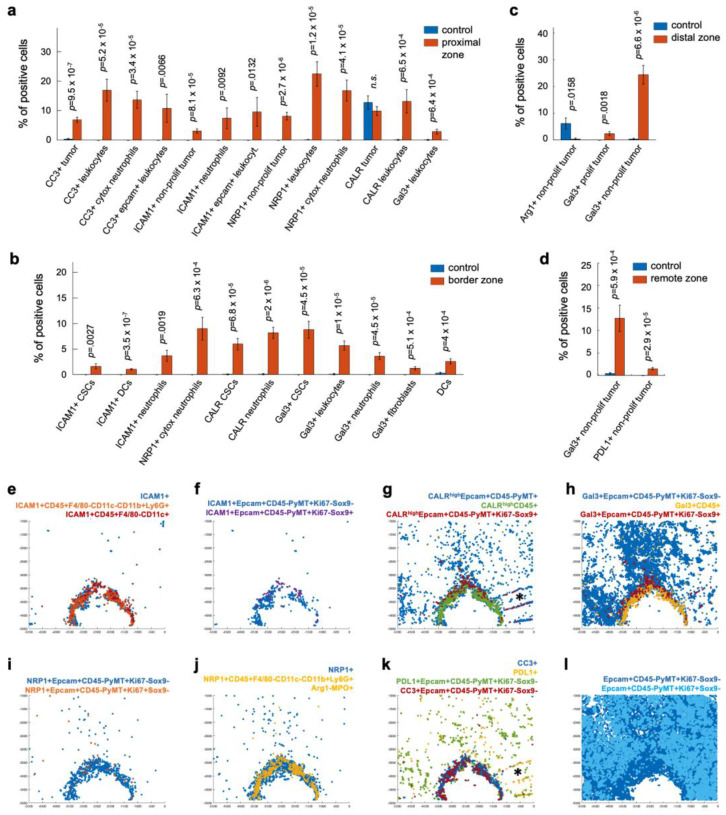
**Quantification of significantly altered cellular phenotypes and their spatial expression in panobinostat-treated tumor samples**. (**a**) Quantification of significantly changed biomarker combinations defining cell-type-specific candidate marker expression in proximal (**a**), border (**b**), distal (**c**), and remote (**d**) regions and their presentation in the XY-coordinate system (**e**–**l**). Late-stage MMTV-PyMT tumors were implanted with panobinostat-loaded microdevices, and the drug was passively released for three days. *n* = 5, 6, 6, and 7 regions for the proximal, border, distal, and remote zones, respectively. Bars are means ± s.e. * folded tumor tissue was excluded from the analysis.

We explored the potential functional roles of these biomarkers in panobinostat-mediated immunogenic cell death by assessing the proximities of the cells expressing them on our standard cell types [1]. Standard cell types are classified by marker combinations in hierarchical gating (Figure 4a–c) and include all major components of tumors and the associated immune and non-immune stroma [1], for example, proliferating tumors (Epcam+CD45-PyMT+Ki67+Sox9-), cancer stem cells (Epcam+CD45-PyMT+Ki67-Sox9+), CD8 T cells (Epcam-CD45+CD3+CD8+CD4-), regulatory CD4 T cells (Epcam-CD45+CD3+CD8-CD4+Foxp3+), dendritic cells (Epcam-CD45+F4/80-CD11c+), cytotoxic N1(-like) neutrophils (Epcam-CD45+F4/80-CD11c-CD11b+Ly6G+Arg1-MPO+), and endothelial cells (Epcam-CD45-CD31+aSMA-). A complete list of the measured standard cell types and defining biomarker combinations is presented in Figure 4c and Supporting Figure S2. By evaluating the candidate biomarker expression on these functionally broad cell populations, we analyzed a total 324 marker combinations on a single-cell basis (Figure 4d). In each zone, we analyzed between five and eight regions of interest, each of which included between 200 and 500 cells, on average (Figure 4e). The total cell counts in the control regions matched those in the panobinostat assay areas (Figure 4e). This semi-supervised spatial cell analysis and classification approach allowed us to identify 200+ marker combinations that were significantly altered between the random control intratumoral regions and one of the panobinostat zones (Figure 4d). The presentation of these markers on lymphoid cells, in general, was a rare event in the intratumoral as well as the assay region. Inversely, the majority of the significant changes appeared within the myeloid cell compartment and the tumor itself (Supporting Figure S2). Some of them are described in detail in Figure 5. We note that only marker combinations appearing in >1% of cells are discussed, as this is a typical threshold used in clinical practice to stratify patients benefiting from PD-1/PD-L1-axis-targeting therapies [36].

We found that the majority of the dying cells (CC3) present in the proximal region were tumor cells and cells expressing both epithelial and leukocyte markers. Cytotoxic neutrophils comprised the largest fraction of the leukocyte population in the proximal region (13.3% of the total cell count, *p* = 3.4 × 10^−5^, Figure 5a,k). These results suggest that neutrophils might mediate cell death both by cytotoxicity (Figure 2a) and well as by dying themselves and causing collateral damage to kill residual cells surrounding them. The latter is similar to acute sterile inflammatory responses [62].

ICAM-1 was broadly expressed, including on non-proliferating tumor cells in the proximal region (Figure 5a,f) and CSCs in the border region (Figure 5b,f), which increased the repertoire of cells typically expressing this marker, such as endothelial cells [38,51], N1 neutrophils (Figure 5a,b,e) [43,44], and licensed DCs (Figure 5b,e) [41,42]. The presence of ICAM-1 in the neutrophil-rich region (Figure 2a) suggests that, early after induction of immunogenic cell death (day 3 of panobinostat exposure), the neutrophils are anti-tumor, N1-polarized, and possibly licensed.

Neuropilin-1 had a very similar pattern to that of ICAM-1, where it was expressed by non-proliferating rather than proliferating tumor cells (Figure 5a,i) and by (cytotoxic) neutrophils in both the proximal and border regions (Figure 5a,b,j). The latter confirmed that neuropilin-1 likely has anti-tumorigenic rather than immune-suppressive (arginase-negative, Figure 2a and Figure 4c) activities during immunogenic cell death. Overall, these results imply that this pleiotropic protein might be a new biomarker for anti-tumor neutrophils early during induced anti-tumor immunity in breast cancer.

**Figure 6:**
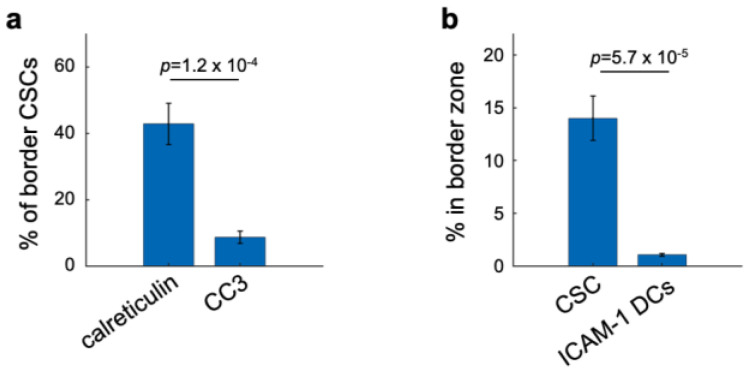
**Functional cell events associated with the cancer stem cell niche.** (**a**) Quantitative comparison of the “eat me” signal, calreticulin, compared to the cell death signal, CC3, within the cancer stem cell (CSC) population in the panobinostat assay border zone. (**b**) Quantitative comparison of CSCs and ICAM-1-positive dendritic cell (DC) abundancy in the panobinostat border zone. Late-stage MMTV-PyMT tumors were implanted with panobinostat-loaded microdevices, and the drug was passively released for three days. *n* = 8 regions containing between 198 and 251 cells. Bars are means ± s.e.

Surprisingly, calreticulin was not significantly expressed on tumor bulk in any of the zones (Figure 5a,g). This was likely because high-calreticulin tumor cells often appeared unspecifically in random control tumor regions independent of panobinostat efficacy (Figure 4d). Instead, the majority of high-calreticulin cells were leukocytes in the proximal panobinostat zone (Figure 5a,g). We observed high-calreticulin cancer stem cells in the border region (Figure 5b,g), suggesting they can be phagocytosed by the surrounding myeloid cells (Figure 5b,e). Out of all CSCs, about 42% were high in calreticulin, while only 9% were CC3-positive (Figure 6a), which is line with our previous observations showing exclusive staining of cancer stem cells and dying cells [1]. These quantitative measurements imply that cancer stem cells are more likely to be eaten/phagocytosed and less likely to die. The remaining question is how this lack of cell death affects the overall anti-tumor immunity specific to CSCs and whether it can be effectively enhanced and/or targeted. Dendritic cells in the CSC-rich “border” region expressed ICAM-1 (about 40% of them; Figure 5b,e), implying that they are prone to be licensed if they express additional antigen-presenting and maturation markers [41,42]. However, there was significantly less of these ICAM-1+ DCs compared to the total CSCs (by 13.2-fold; Figure 6b). These CSC-associated results indicate that effective therapy responses might have both qualitative as well as quantitative characters. More importantly, targeting CSCs both (i) through direct killing as well as through immune modulation and (ii) by qualitative and quantitative means might be required to induce long-term breast cancer control.

PD-L1 expression was observed to be panobinostat-specific and a rare event unique to non-proliferating tumors in regions remote from the well (Figure 5d,k).

Galectin-3 was expressed on multiple cell types, depending on the zone: leukocytes in the proximal zone (Figure 5a,h), CSCs and fibroblasts in the border zone (Figure 5b,h), and non-proliferating tumor cells in the distal and remote (Figure 5c,d,h) regions, respectively. These measurements imply that galectin-3 is spatially linking all observed phenotypes and thus might play a critical and pleiotropic role in induced anti-tumor immunity in BC. We note that all observed phenomena were limited to non-proliferating tumor regions (Figure 5l and Supporting Figure S3).

Overall, these spatial single-cell evaluations of biomarkers’ functions complement the previously identified cellular events describing the resistant cancer stem cell niche in panobinostat-induced immunogenic cell death [1].

### 3.5. Spatial Clustering of Marker Combinations Serves as a Treatment-Specific Biomarker with Predictive Value 

**Figure 7:**
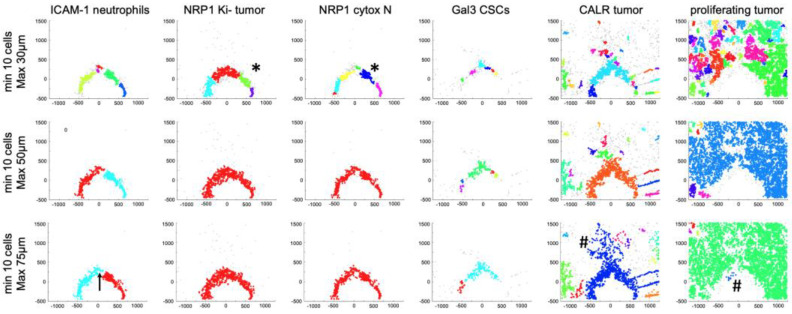
**Distance-based clustering of panobinostat-induced cell types in situ.** Clusters were identified by the presence of a minimum of ten cells within maximum distance ranges of 30 (**top**), 50 (**middle**), and 75 (**bottom**) μm for the depicted cell types (titles). Individual cells are shown as gray points; treatment-specific clusters are depicted with a randomized color. Clusters within the maximum distance range merged and share the same color. Signs “*” and “#” mark fragmented or treatment-unspecific clusters, respectively. Coordinate [0,0] identifies the drug source. An arrow (bottom left) shows the direction of drug release.

To identify hotspots of interest in an unbiased, automated, and biology-driven way, we performed a distance-based cluster analysis [1], implementing the biomarker combinations defined above. This cluster analysis did not require the number of clusters to be known beforehand, as is normally the case in most unsupervised clustering techniques. Instead, the cell clusters were detected if the minimum number of cells were present within a defined maximum distance range in the XY-coordinate space (Figure 7). The Euclidean distance between a set of cells was measured. By mapping the locations of specific cell types where ten or more cells occurred within 30, 50, and 75 μm diameters, we showed that these cells (by biomarker combinations, Figure 4c) clustered together in response to the panobinostat stimulus, and such clusters appeared proximal to the panobinostat well and did not appear in remote regions (Figure 7, colored vs. gray dots in the XY space). All three clustering strategies were effective to detect treatment-specific clusters of presented cells. However, detecting a cluster within a 30 μm diameter range was less specific, as clusters could be fragmented (Figure 7, *). Detecting clusters within a 75 μm diameter range also occasionally resulted in an unspecific formation of clusters in regions where significant alterations were not measured (Figure 7, #). In summary, this analysis suggests that specific spatial cell associations appearing in clusters during the treatment response might serve as novel in situ biomarkers with predictive value for anti-PD-1 immunotherapy efficacy in breast cancer.

## 4. Discussion

PD-L1 expression in tumors was the first candidate molecular marker identified in association with the objective response in patients treated with anti-PD-1 immunotherapy [8,63]. However, several clinical trials showed only moderate efficacy of ICB in patients with PD-L1 expression [64], and other studies have shown durable ICB efficacy in patients with PD-L1-negative tumors [65,66]. The identification of mechanism-based predictive biomarkers that could be used to guide treatment management decisions remains challenging. Implementing the MIMA tool, we were also unable to measure consistent PD-L1 expression, as detected by IHC. In the MMTV-PyMT mouse model of breast cancer, the local delivery of panobinostat led to specific PD-L1 enrichment in only one out of three replicates [1], further questioning the predictive value of this approach. The mechanisms underlying PD-L1 upregulation are broad and complex, and thus it is not surprising that IHC-based measurements are difficult to interpret and are often inadequate [66].

Tumor-infiltrating lymphocytes are thought to reflect an ongoing anti-tumor immune response. The IHC-based Immunoscore, which takes into account the density of CD8 and CD3 within the tumor and the invasive margin, has been shown to have predictive value for anti-cancer therapy responses [67]. However, due to the considerable heterogeneity and possible molecular subtype/host immune system association, TILs might have limited predictive value in BC [8].

In this study, we provide a deep literature review summarizing IHC-based biomarkers that might be important determinants of induced anti-tumor immunity and ICB efficacy. Using MIMA, were able to precisely and comprehensively quantify their presence in response to a controlled bolus intratumor delivery of panobinostat and present their spatial expression with respect to the myeloid subsets playing a major role in immunogenic cell death in breast cancer [1]. We also systematically evaluated which cell types and states specifically express the candidate probes, which eventually described their likely role in ICD, and found new biomarkers in the form of marker combinations as well as specific spatial cell associations. Among the most interesting biological findings, we further affirm that neuropilin-1 might be a novel biomarker of anti-tumor and/or cytotoxic neutrophils early during immunogenic cell death in BC. One future consideration is to validate this observation and specificity with an alternative antibody from a different vendor. This would be highly useful because neuropilin-1 has recently been associated with pro-tumorigenic rather than anti-tumor processes. Intratumoral NRP1+ regulatory T cells were described to correlate with poor prognosis in melanoma and head and neck squamous cell carcinoma, where NRP-1 functioned to maintain T_regs_ stability [68]. Another study showed that neuropilin-1 can actively bind TGFβ1 and is involved in epithelial-to-mesenchymal transition processes in pancreatic ductal adenocarcinoma [69]. The likely anti-tumor function of the neuropilin-1 we observed might be breast-cancer-specific and is more in line with the older literature describing its essential role in the initiation of the primary immune response (Tordjman et al., 2002) [54]. Therefore, neuropilin-1 might have a dual, contextual, and tumor-type-specific immunomodulatory role in cancer and immunotherapy. Its function in the licensing of myeloid cell subsets will have to be further tested using the expression of activation, maturation, and antigen-presenting markers such as CD80, CD86, Class I and II major histocompatibility complex (MHC-I, -II), and ICAM-1. Other interesting results were linked to galectin-3 expression. This protein was expected to be expressed on infiltrating phagocytic macrophages, as previously described in the MMTV-PyMT model after class II HDAC inhibitor application [46]. However, our single-cell in situ measures showed the protein to be expressed broadly on non-proliferating tumor cells, neutrophils, DCs, fibroblasts, and even cancer stem cells. The latter suggests that galectin-3 might be used to enrich cancer stem cell pools in treated mammary carcinoma. The divergence of our data and the published results [46] may derive from the fact that (i) we are not testing the same drug (panobinostat is a pan-HDAC inhibitor targeting multiple HDAC classes and might trigger different immune infiltration), (ii) we did not image the tumors at the same timepoint (day 3 vs. day 5 responses), and (iii) the reference study did not measure in situ drug responses with mIHC on a single-cell basis. Moreover, ICAM-1 was expected to be associated with perivascular regions [15,39], but majority of the panobinostat-induced expression was within the tumor and neutrophil compartments. It is possible that tumor cells themselves and neutrophils (and to smaller extent DCs, Figure 5b,e) are important initiators of induced anti-tumor immunity in breast cancer. On the other hand, systemic therapies might induce a different cell type enrichment or some previously identified phenotypes might have been misclassified, as multiplex immunostaining and single-cell classification using a set of probes is a relatively new technology [1,27,28], especially for mouse specimens [1]. In this sense, the MIMA tool has both disadvantages and advantages. On one hand, it is limited to early and local intratumoral drug response events; on the other hand, it provides direct, precise, and controllable perturbations that lead to intratumoral evidence of drug effects and thus allows researchers to define and refine new and old cell biology insights based on direct in situ measurements. It also highlights the strength of multidimensional single-cell analysis and flow-cytometry-like hierarchical gating. The MIMA cell classification approach [1] is broadly defined and allows researchers to narrow down a target phenotype by cell type (e.g., tumor or neutrophil) as well as by differentiation or functional cell state (e.g., cancer stem cells or anti-tumor cytotoxic neutrophils). The underlying biology is derived from the computational processing of complex dynamics between the drug, the tumor, and the host tumor microenvironment, and overall the strategy helps to interpret the data into biologically meaningful findings.

Previously, we showed that the local delivery of panobinostat induced a unique enrichment of an unusual population of antigen-presenting neutrophils suggestive of the induction of ICD. ICD induction was subsequently confirmed by demonstrating the recruitment of CD8 T cells inside the tumor bed using a vaccination study as well as by showing synergistic effects with anti-PD-1 in whole mouse studies. Cancer stem cells emerged at the interface of the immune-infiltrated ICD region and tumor, suggestive of an important mechanism of resistance [1]. In the current study, we further described and quantified the cellular mechanism of action of panobinostat in breast cancer, including that (i) only about 10% of CSCs died from drug exposure; (ii) 42% expressed the “eat me” signal, calreticulin; and (iii) myeloid cells are prone to be licensed but are present in limited amounts compared to the total CSCs in their niche. Additionally, in the cell-death-rich region, the majority of the high-calreticulin cells were myeloid cells rather than tumor cells. Calreticulin on and secreted by macrophages has been reported to play an essential role in adjacent programmed cell removal, with Toll-like receptors and Bruton’s tyrosine kinase pathways being involved [70]. Similar to our results, the calreticulin expression on tumor cells was not essential in the process. Instead, the downregulation of the antiphagocytic CD47 “don’t eat me” signal on tumor cells was deterministic for phagocytosis by macrophages [70], and thus the measurement of this biomarker could be implemented in a future readout. In general, supplementary integrations of the MIMA system with other spatial and single-cell -omic technologies could potentiate a more detailed molecular characterization of the panobinostat-induced ICD in vivo. In hepatocellular carcinoma tumor xenografts in nude mice, panobinostat induced non-canonical apoptotic cell death involving endoplasmic reticulum stress [71]. In non-small-cell lung cancer and head and neck squamous cell carcinoma, panobinostat potentiated the killing of persistent senescence cells that emerged after a standard chemotherapy treatment [72]. This suggests that the cell death and stress responses induced by panobinostat might be heterogeneous, and its function in senescence-mediated resistance (senolytics) should be explored further.

Overall, it will be interesting to see how the local marker- and cell-specific clustering analyses translate into the human setting and to other localized and systemic therapy applications. Here, we provide a relatively broad catalog of markers and cell types that, if identified in clusters, might reflect ongoing immunogenic cell death and might be predictive of PD-1 pathway blockade efficacy in breast cancer. The readout is IHC-based and thus could be applied to future as well as historical FFPE samples. The general approach can be applied to other drugs and candidate predictive biomarkers, so MIMA may be generally useful for identifying predictive cell-based and mechanism-driven biomarker patterns.

## Figures and Tables

**Table 1:** IHC-based markers previously linked to ICD and/or ICB.

Biomarker	Full/Alternative Name and Literature	Reference [37]
ICAM-1	Intracellular adhesion molecule 1	*P13597*
	Increased expression in the vascular and perivascular spaces during ICD induced by ionizing radiotherapy in BC [15] or by cabozantinib in prostate cancer [38], respectively. Absent tumor expression is prognostic of lymphatic spread in early lung cancer [39]. Melanoma patients responding to anti-PD-1 immunotherapy have higher ICAM-1 on circulating monocytes [40]. ICAM-1 is a biomarker of licensed myeloid cells [41,42] and anti-tumor neutrophils [43,44].	*Krombach et al., 2019; Patnaik et al., 2017; Passlick et al., 1996; Krieg et al., 2018; Vonderheide, 2018; Lanzavecchla, 1998; Fridlender et al., 2009; Shaul et al., 2016*
CALR	calreticulin	*P14211*
	Pre-apoptotic translocation of calreticulin to the cell surface is an indicator of induced immunogenic cell death and serves as an “eat me” signal for phagocytosis by dendritic cells [21] and neutrophils [24]. Calreticulin also facilitates the folding of class I major histocompatibility complex (MHC-I) molecules, thereby affecting antigen-presenting machinery and T-cell responses [45].	*Obeid et al., 2007; Garg et al., 2017; Raghavan et al., 2013*
Gal-3	galectin-3, also known as Mac-2	*P16110*
	A class II HDAC inhibitor shown to be synergistic with anti-PD-1-immunotherapy-induced galectin-3 expression in mammary carcinoma, as measured by IHC [46]. Galectin-3 targets and activates autophagy [47], which is required for the immunogenicity of cell death [48]. Galectin-3 is an inducible “danger signal” molecule for innate immunity [49] and activates extravasated but not peripheral blood neutrophils [50]. Exogenous galectin-3 and ICAM-1 were shown to be involved in the slow rolling of leukocytes [51] and their recruitment into the tissue from the inflamed microcirculation [52].	*Guerriero et al., 2017; Chauhan et al., 2016; Michaud et al., 2011; Sato and Nieminen, 2002; Karlsson et al., 2018; Yang et al., 2005; Gittens et al., 2018*
MPO	myeloperoxidase	*P11247*
	A profound anti-tumor response to cabozantinib was accompanied by cytotoxic (MPO) neutrophil (Ly6G) infiltration in prostate cancer. In this study, cabozantinib induced tumor cell death in vivo, and ex vivo the therapy induced HMGB-1 release to the supernatant, as measured using ELISA, which was suggestive of ICD induction [38].	*Patnaik et al., 2017*
NRP-1	neuropilin-1	*P97333*
	In breast cancer cells, neuropilin-1 is involved in cross-presentation and is essential for the killing of neutrophil elastase peptide specific cytotoxic T-cell [53]. Early studies on neuropilin-1 focused on parallels of the immune and nervous systems and showed that the protein can be expressed on DCs and resting T cells. Neuropilin-1 mediated clustering between these two populations, the polarization of the protein upon contact, and proliferation of T cells, suggesting that it is essential for the initiation of the primary immune response [54].	*Kerros et al., 2017; Tordjman et al., 2002*
CC3	Cleaved caspase-3	*NA*
	Well-established biomarker of cell death	*multiple*
PD-L1	Program (cell) death ligand 1, B7 homolog 1 (B7-H1)	*Q9EP73*
	Established biomarker of ICB efficacy	*multiple*

## Data Availability

All data that support the findings of this study are available within the article and its Supplementary Information or from the corresponding authors upon reasonable request.

## Supplementary Materials

The following supporting information can be downloaded at https://www.mdpi.com/article/10.3390/cells12020308/s1, Supporting Figure S1: Multiplex IHC (mIHC) of candidate markers using positive control FFPE tissue; Supporting Figure S2: Marker combinations and their spatial quantification at the panobinostat drug release site; Supporting Figure S3: Magnified view of cell types in XY-coordinate space.
