# Supplementary material for: Panobinostat Induced Spatial In Situ Biomarkers Predictive of Anti-PD-1 Efficacy in Mouse Mammary Carcinoma
Source: Cells. 2023 Jan 13;12(2):308. doi: 10.3390/cells12020308 (PMC9856407; doi:10.3390/cells12020308)
Supplement: Supplementary file 1 [file cells-12-00308-s001.zip › cells-2038708-supplementary.pdf]

Panobinostat induced spatial *in situ* biomarkers predictive of anti-PD-1 efficacy in mouse mammary carcinoma

Zuzana Tatarova [1,2,3,*], Dylan C. Blumberg [1,3], AeSoon Bensen [1,4], Lisa M. Coussens [2,4], Gordon B. Mills [5], Oliver Jonas [3,*]

Supporting Fig. 1

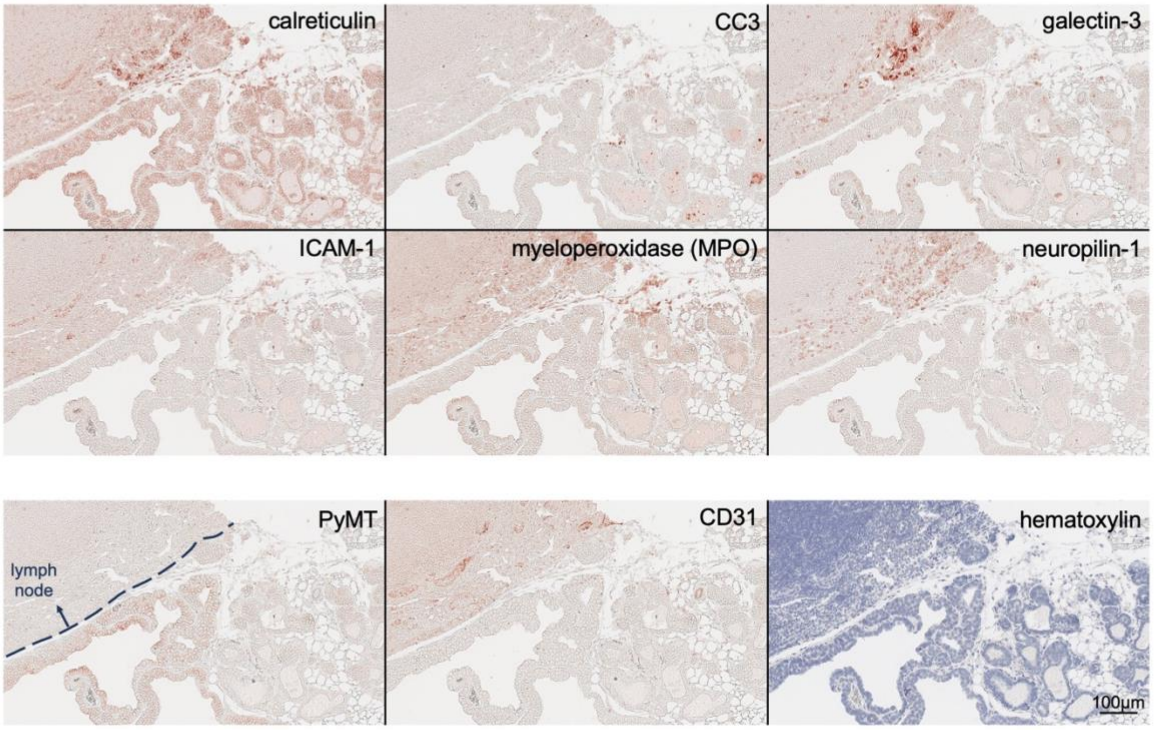

Supporting Figure S1. Multiplex IHC (mIHC) of candidate markers using positive control FFPE tissue. Brightfield images of stated markers using mIHC (red signal from 3-amino-9ethylcarbazol, AEC, precipitate). FFPE tissue section containing an early tumor (<0.5cm in the longest dimension) from MMTV-PyMT mouse model of breast cancer and adjacent lymph node (border depicted by dashed line; bottom left). Hematoxilin staining; bottom right image. Scale bar; shown.

Supporting Fig. 2

Epcam+CD45-
Epcam+PyMT+
Epcam+PyMT+Ki67+Sox9-
Epcam+PyMT+Ki67-Sox9+
Epcam+PyMT+Ki67+Sox9+
Epcam+PyMT+Ki67-Sox9-
Epcam-CD45+
Epcam-CD45+CD3+
Epcam-CD45+CD3+CD4+CD8-
Epcam-CD45+CD3+CD4+Foxp3+
Epcam-CD45+CD3+CD4+Foxp3-
Epcam-CD45+CD3+CD4-CD8+
Epcam-CD45+CD3+CD8+GzmB+
Epcam-CD45+CD3+CD8+GzmB-
Epcam-CD45+CD3+CD4+CD8+
Epcam-CD45+CD3+CD4-CD8-
Epcam-CD45+CD45+F480+CD11c-
Epcam-CD45+CD45+F480+CD11c-CSF1R+
Epcam-CD45+CD45+F480+CD11c-CSF1R-
Epcam-CD45+CD45+F480-CD11c+
Epcam-CD45+CD45+F480+CD11c+
Epcam-CD45+CD45+F480-CD11c-
Epcam-CD45+CD45+F480-CD11c-CD11b-Ly6G+
Epcam-CD45+CD45+F480-CD11c-CD11b+Ly6G-
Epcam-CD45+CD45+F480-CD11c-CD11b+Ly6G+
Epcam-CD45+CD45+F480-CD11c-CD11b+Ly6G+Arg1+MPO-
Epcam-CD45+CD45+F480-CD11c-CD11b+Ly6G+Arg1-MPO+
Epcam-CD45+CD45+F480-CD11c-CD11b+Ly6G+Arg1+MPO+
Epcam-CD45+CD45+F480-CD11c-CD11b+Ly6G+Arg1-MPO-
Epcam-CD45+F480-CD11c-CD11b-Ly6G-
Epcam-CD45-
Epcam-CD45-aSMA-CD31+
Epcam-CD45+aSMA+CD31-
Epcam-CD45-aSMA+CD31+
Epcam+CD45+

proximal	border	distal	remote
0.07108389	0.04348244	0.0013495	0.00400447
0.07108389	0.04348244	0.0013495	0.00400447
0.00579165	0.00192166	0	0
0.00905914	0.01236828	0	0
0	0.0007215	0	0.0010989
0.0562331	0.02847101	0.0013495	0.00290557
0.16583959	0.0832057	0.00032237	0.00032175
0	0.00060024	0	0
0	0	0	0
0	0	0	0

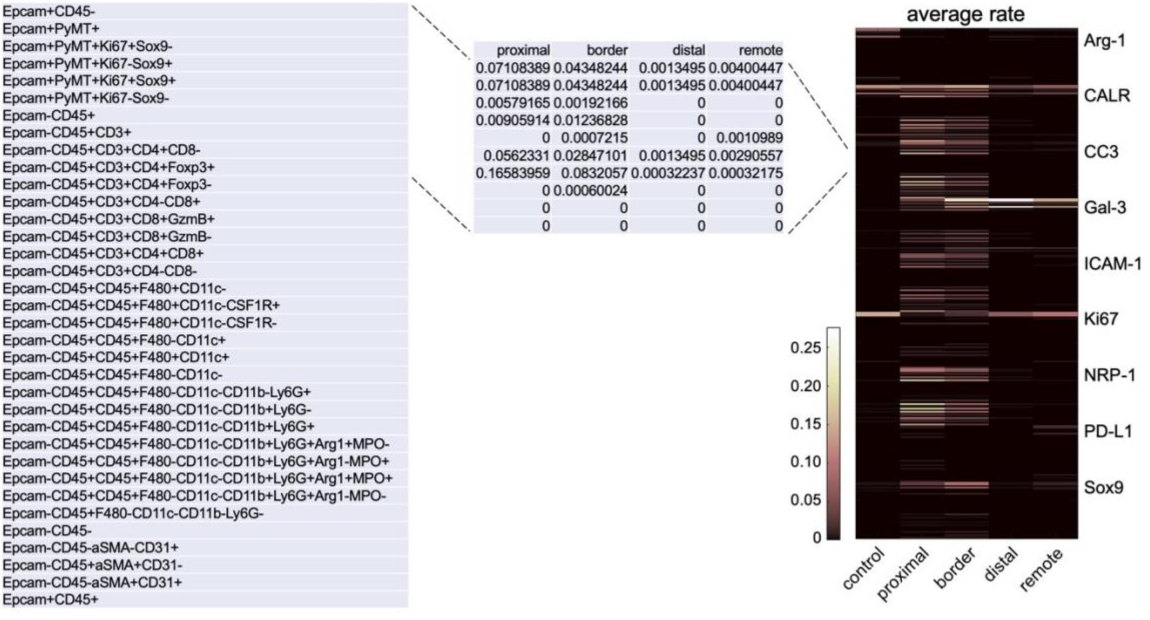

Supporting Figure S2. Marker combinations and their spatial quantification at the panobinostat drug-releasing site. Expression of each candidate ICD marker was measured within “standard cell type” population as defined by marker combination; left list. Zoomed view of quantitative presentation of average cell rates in different panobinostat zones; middle table. Quantification of all 324 marker combinations per panobinostat zone (x-axis) presented in form of a heatmap; right. Average rate and colorbar; presented.

Supporting Fig. 3

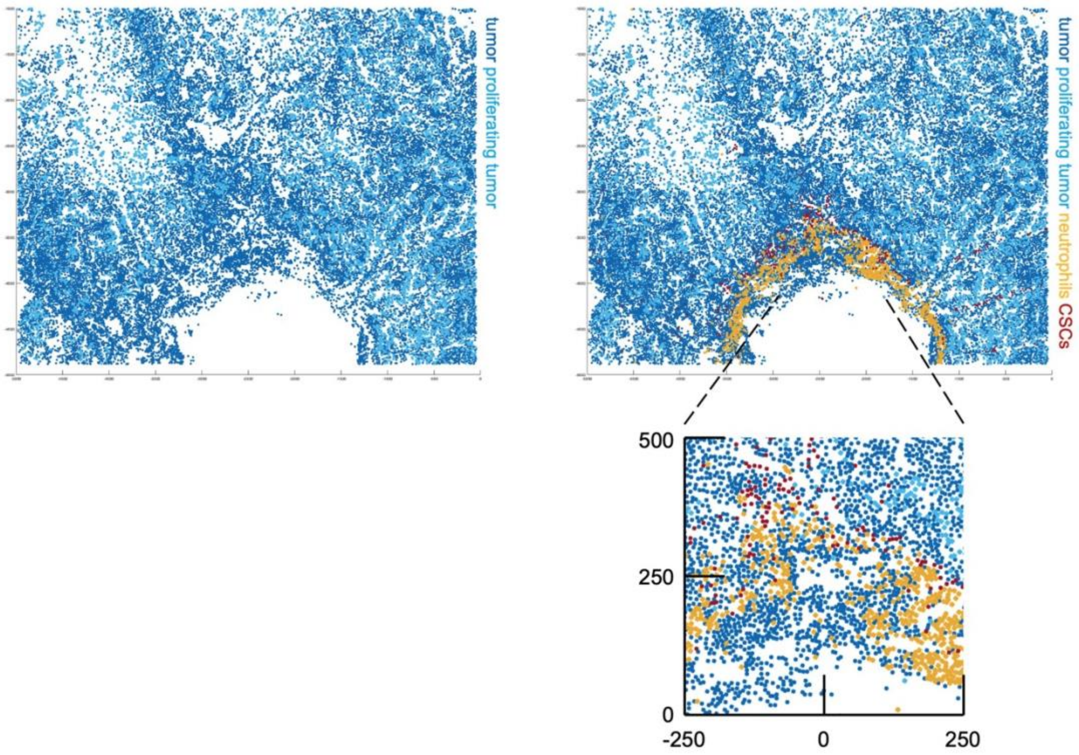

Supporting Figure S3. Zoom view of cell types in XY coordinate space. Each dot represents a single cell as color-coded on the right. Neutrophil and cancer stem cells (CSCs) are localized into non-proliferating tumor regions; top. Magnified view; bottom right.
